# Unveiling Unwritten Curricula: Enhancing Junior Faculty Development in Academic Emergency Medicine

**DOI:** 10.5811/westjem.50856

**Published:** 2026-06-28

**Authors:** Abagayle Bierowski, Casey Morrone, Erin Hoag, Ridhima Ghei, Michael Pasirstein, Julie Blaszczak, Dimitrios Papanagnou

**Affiliations:** * Thomas Jefferson University, Department of Emergency Medicine, Philadelphia, Pennsylvania; † Sidney Kimmel Medical College, Philadelphia, Pennsylvania; ‡ Wake Forest University, Department of Emergency Medicine, Winston-Salem, North Carolina; § Wake Forest University, School of Medicine, Winston-Salem, North Carolina; ¶ University of Michigan Medical School, Ann Arbor, Michigan; || NYU Langone Hospital, Department of Emergency Medicine, Mineola, New York; # University of Michigan, Department of Family Medicine, Ann Arbor, Michigan

## Abstract

**Introduction:**

The transition from resident to junior faculty in academic emergency medicine (EM) may be shaped not only by formal training and institutional policies but also by the “unwritten curriculum,” a set of norms and expectations embedded in institutional culture. However, the unwritten curriculum and its potential to impact junior faculty development remains largely unexplored. Little is known about how junior faculty perceive, experience, and navigate these informal expectations, or the formal and informal structures that influence this process. In this study we aimed to explore junior faculty members’ experiences with the unwritten curriculum in academic EM, with a focus on how they interpret and navigate institutional norms and identify supports and barriers encountered during their early faculty development.

**Methods:**

Within their first five years, EM faculty at academic institutions distinct from their residency training sites completed an anonymous, iteratively developed survey designed to explore experiences with the unwritten curriculum, informed by Schlossberg’s transition theory framework. The primary analytic focus was identification of themes describing how junior faculty perceive and navigate implicit institutional norms. Open-ended responses underwent thematic analysis using a structured codebook, applied by two independent reviewers with consensus-based coding and adjudication by a third when disagreements arose.

**Results:**

A total of 35 junior faculty members completed the survey. All participants indicated influence of the unwritten curriculum on their professional development. Major themes identified include the unspoken importance of mentorship and peer guidance; implicit expectations and norms surrounding engagement, productivity, and visibility; work-life integration; cultural adjustment; scholarly output; and gaps in onboarding, feedback, and role clarity. Participants emphasized challenges in navigating departmental politics, understanding hierarchical nuances, and balancing unspoken expectations for committee involvement and informal social participation. Additionally, unclear feedback processes, inconsistent evaluation expectations, and cultural norms for active engagement were noted as barriers to integration and professional growth.

**Conclusion:**

Junior faculty in academic EM described the unwritten curriculum as a meaningful influence on their early faculty experience. Within this sample, participants highlighted mentorship, feedback transparency, and structured faculty development as potential mechanisms to support navigation of implicit expectations during the transition to junior faculty. These findings reflect individual perceptions rather than program characteristics and should be interpreted within the context of the study’s scope.

## INTRODUCTION

The transition from resident to junior faculty in academic emergency medicine (EM) extends beyond formal training and technical proficiency. Junior faculty must also navigate the “unwritten curriculum”—a set of implicit norms, values, and expectations. While formal curricula provide structured education in teaching and clinical practice, the unwritten curriculum operates beneath the surface, embedded within institutional culture, workplace dynamics, and professional interactions. The unwritten curriculum is conceptually distinct from the hidden curriculum, which has been extensively examined in the medical education literature and refers to institutional-level transmission of implicit values and norms.^1^ Because junior faculty play a critical role in academic growth and innovation, the success of academic health centers is closely tied to how effectively they are recruited, supported, and advanced.^2^

Studies suggest that residents and newly graduated attending physicians actively engage with, shape, and attempt to influence these informal learning processes as they navigate their professional development.^1^ This underscores the need for structured mentorship and early career guidance to help junior faculty assimilate and contribute meaningfully to their academic environments. National needs assessments of EM faculty development echo this point, revealing gaps in education-related programming, persistent deficits in mentorship and wellness resources, and varying levels of early career satisfaction, all of which highlight the need for more tailored and intentional support.^3^,^4^ Faculty retention is also linked to implicit workplace expectations, with breaches of unwritten contract obligations contributing to departures.^5^ These findings suggest that the unwritten curriculum extends beyond faculty training, influencing career satisfaction and long-term engagement in academic EM.

Existing literature explores the *hidden* curriculum in medical education, which transmits attitudes and values through what is (or isn’t) taught and has been shown to influence professional development.^1^,^6^,^7^ The hidden curriculum is often viewed as a negative, institutional-level concept involving unintended or obscured lessons.^8^,^9^ Related constructs describing implicit or informal learning processes have also been used to characterize norms and expectations conveyed through culture, behavior, and professional socialization. By contrast, the *unwritten* curriculum has been less consistently defined and consists of implicit norms and expectations that may influence faculty development but are not formally documented or explicitly taught. Rather than containing intentionally obscured information, the unwritten curriculum instead represents a series of implicit norms and expectations that faculty must piece together to fully assimilate into academia.

In this study, we use the term *unwritten curriculum* to describe this subset of informal learning processes as they are experienced during early faculty transitions. This unwritten curriculum remains largely unexplored in the transition to faculty life, leaving new educators to navigate implicit expectations through trial and error, which may hinder career growth, scholarly productivity, and institutional integration. Because faculty who remain at their residency training institution may already be familiar with local norms and expectations, this study focused on junior faculty whose first academic appointment was at a different institution to better capture active navigation of the unwritten curriculum and reduce bias related to prior institutional socialization.

**Population Health Research Capsule**

What do we already know about this issue?

*Junior EM faculty report challenging transitions, but structured onboarding and mentorship models remain poorly defined*.

What was the research question?

*What gaps exist in onboarding and support during junior EM faculty transition?*

What was the major finding of the study?

*Only 35% rated first-year support as good/excellent; 64% reported unclear role expectations*.

How does this improve population health?

*Identifying onboarding gaps may improve faculty retention, well-being, and stability of the emergency care workforce*.

This study explores how junior faculty experience, interpret, and navigate the unwritten curriculum in academic EM. The primary objective was to identify recurring themes describing junior faculty perceptions of implicit norms and expectations during the transition to their first academic faculty role. Secondary objectives included examining the formal and informal strategies junior faculty use to navigate these expectations and identifying perceived facilitators and barriers to early faculty integration and development.

## METHODS

### Study Design and Conceptual Framework

Faculty participated in an anonymous online survey through REDCap (Research Electronic Data Capture), a secure, web-based software platform designed to support data capture for research studies.^10^,^11^ We selected a survey-based qualitative design to allow anonymous participation from junior faculty across institutions and to encourage candid reflection on implicit norms and expectations. Open-ended qualitative questions were designed to address the four key domains of adaptation in Schlossberg’s transition theory framework.^12^ (See Appendix B for complete survey.) We selected Schlossberg’s transition theory because it provides a structured framework for examining how individuals experience and adapt to professional transitions. The framework identifies four key domains influencing an individual’s ability to cope with transition: situation (circumstances); self (personal attributes); support (resources); and strategies (coping methods). These domains informed the study design by guiding the areas of inquiry related to junior faculty transition experiences in academic EM.

We developed the survey using tenets of constructivism, which posits that individuals build meaning based on their personal experiences and interactions with their environment. This perspective shaped the overall study design by prioritizing participants’ subjective interpretations of institutional culture, professional identity, and transition experiences. This informed our inclusion of open-ended questions to allow participants to express individualized perspectives and reflections on their transitional experiences. Furthermore, the structure of the survey encouraged participants to reflect on how their identity, values, and institutional context shaped their perception of professional growth and challenges.^13^

### Study Setting and Population

We recruited EM faculty within their first five years of practice at academic institutions distinct from their residency training sites using a combination of purposive and convenience sampling. Eligible participants were identified through professional networks, national EM education communities, and snowball sampling. Participants were invited via direct email outreach; recruitment occurred over a defined period and included an initial invitation with a brief study description and survey link, followed by one reminder email. Recruitment targeted individuals who fit our inclusion criteria: university-appointed EM residency program faculty with ≤ 5 years of post-residency experience, practicing at academic hospitals unique from their residency training program.

Eligibility was confirmed through self-report screening questions embedded at the beginning of the survey, including confirmation of years in practice, current academic appointment, and whether the participant’s current institution differed from their residency training site. Participants who did not meet inclusion criteria were not permitted to proceed with the survey. Recruitment continued until thematic saturation was reached—defined as the point at which no new themes or insights were emerging from the data. This approach is consistent with qualitative research standards for exploratory, open-ended, survey-based studies.^14^ All participants were aware of the investigators’ goals for the project and were offered the opportunity to download a copy of their responses after survey completion, although no post-submission edits were permitted.

### Survey Development

In the absence of any pre-existing surveys appropriate for modification, the survey instrument (Appendix B) was developed iteratively by the research team, guided by existing literature on junior faculty development, transition challenges, and professional identity formation in academic medicine. It was reviewed by two EM faculty development and medical education experts to assess content validity, clarity, and relevance. The instrument was then pilot-tested with four early-career EM faculty who met inclusion criteria and then were excluded from the final study sample. Their feedback led to revisions in wording, layout, and flow, ensuring alignment with the conceptual framework and study objectives. While the survey was not formally validated through psychometric testing, expert input and pilot testing supported face validity and informed content refinement consistent with the conceptual framework.

The survey included five demographic questions followed by 17 content questions: 10 open-ended; 5 Likert-scale items; and 2 open-text fields in a conclusion section. Content questions were grouped into five thematic sections: perceptions of the unwritten curriculum, and the four domains of Schlossberg’s transition theory (situation, self, support, and strategies). Each domain-based section included two open-ended questions and one Likert-scale question. A final concluding section invited participants to reflect on their overall transition experience and provide any additional comments they felt were relevant. Questions were presented in a fixed order to preserve conceptual coherence and minimize participant confusion. To ensure human (non-bot) responses, two simple challenge questions were embedded within the survey, prompting participants to perform basic tasks (eg, “four plus four”). Given the open-response format, instructions encouraged participants to respond freely without requiring a minimum word count.

### Ethical Considerations and Reporting Standards

We obtained ethical approval from two institutional review boards (iRISID-2024-3224; HIRB00019708). Participants were invited to voluntarily complete an anonymous online REDCap survey, with consent implied by submission. No identifying information was collected to maintain confidentiality. Participants were not asked to provide feedback on the findings following study completion. This study adhered to the Standards for Reporting Qualitative Research, a synthesis of recommendations to ensure methodological rigor and transparency in the design, data collection, and reporting of qualitative findings (Appendix C).^15^

### Research Team and Reflexivity Statement

The research team for this project consisted primarily of emergency physicians, along with one family medicine physician who contributed to project conception, data analysis review, and final manuscript preparation. All team members (two male, five female) held MD degrees, with additional credentials including an MEHP, an MPH, and an EdD, reflecting diverse expertise. The research team represented two academic institutions. Most team members were junior faculty with strong interests and backgrounds in medical education, while three researchers were senior faculty who brought additional leadership and experience to the project, and one was an expert on qualitative research. Six of the seven authors trained at institutions different from their first academic job. We report no conflicts of interest and received no external funding.

### Outcomes

The primary outcome of the study was thematic characterization of junior faculty perceptions and experiences related to the unwritten curriculum during early academic career transitions.

### Data Analysis

We used computer-assisted qualitative data analysis software to review and analyze all qualitative data. Narrative analysis was based on content analysis, a qualitative method for classifying and categorizing textual information.^16^ Given the limited amount of literature on the unwritten curriculum particularly within academic medicine, categories were derived iteratively through close reading of textual data rather than through external thematic imposition. Two independent reviewers randomly reviewed and openly coded a subset of 50 randomly generated narrative responses. Following this, the lead researcher (INTIALS) developed a tentative codebook including code definitions and direct narrative quote examples. All 385 narrative responses were extracted and uploaded into Dedoose v9.0.107 (SocioCultural Research Consultants, LLC, Los Angeles, CA), a cloud application for managing, analyzing, and presenting qualitative and mixed method research data.^1^ We used the software to organize and manage narrative responses, apply manual codes, and facilitate code-based retrieval and analysis.

Two coders reviewed all responses, iteratively revising the codebook throughout textual content coding as appropriate. Coding discrepancies were reviewed collaboratively, and any disagreements that could not be resolved through discussion were adjudicated by a third member of the research team. The final codebook was limited to fewer than 20 codes, a strategy commonly used in content analysis. Not all reviewed comments were assigned a code; responses that were off topic, ambiguous, or unrelated to the study aims were excluded from thematic coding. Once coding was complete, we summarized content for each code and extracted quotes to include in the results reporting. Thematic analysis was conducted both for the survey’s narrative responses as a whole and separately for each individual question.

## RESULTS

A total of 35 junior faculty members completed the survey. We collected demographic data (age range, sex, and practice location) to examine the diversity of representation of individuals and regions (Table 1). Participants represented a range of academic environments, including university-affiliated tertiary-care centers, safety-net hospitals, and hybrid community-academic programs, spanning both 3- and 4-year EM residency models. While most participants had educational or clinical leadership roles, their scholarly interests varied, with some pursuing research-oriented careers and others focused on education, administration, or quality improvement.

**Figure 1:**
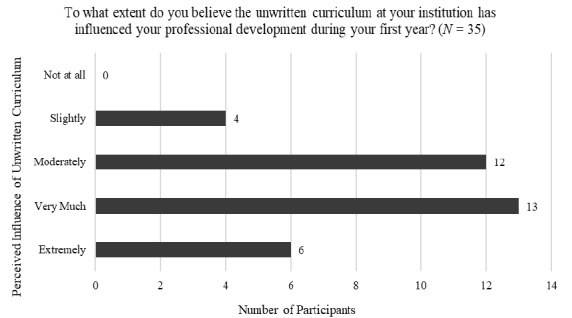
Perceived influence of the unwritten curriculum on professional development among junior academic emergency medicine faculty during their first year.

**Figure 2:**
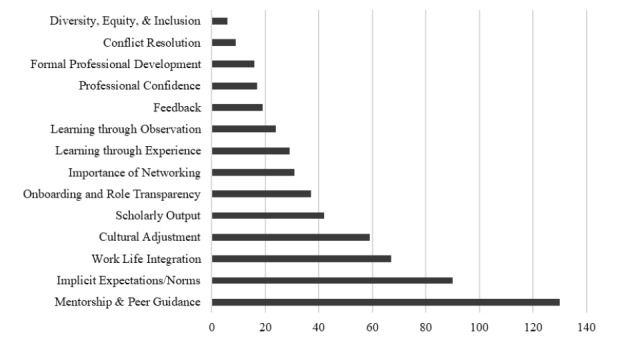
Distribution of thematic codes related to the unwritten curriculum across open-ended survey responses from junior academic emergency medicine faculty.

All 35 participants indicated that the unwritten curriculum had some degree of influence on their professional development during their first year as a faculty member (Figure 1). Figure 2 depicts the distribution of thematic codes across narrative responses. The six most salient themes are presented below in order of prevalence. Additional themes and illustrative quotations are summarized in Appendix A.

### Mentorship and Peer Guidance

Mentorship and peer guidance emerged as the most salient theme in qualitative responses. Participants consistently emphasized the importance of access to both formal and informal mentors and the value of peer guidance in navigating their challenges as a new academic faculty member.

#### Formal Mentorship

Most participants spoke of the importance of departments using deliberate formal mentorship programs to better support junior faculty. One wrote, “Mentorship from senior faculty members has been invaluable in guiding my career path and providing insight into department culture.” Respondents yearned for senior faculty mentors not only with similar interests but also ideally with a nonjudgmental attitude and one “that works a similar clinical burden.” Several suggested that departments create formal mentorship programs pairing junior faculty with experienced mentors to “help clarify the unwritten rules and expectations.” Multiple participants also requested more frequent check-ins with formal mentors or senior faculty, particularly during their first year, citing performance metrics and clinical statistics as areas of interest to discuss.

#### Informal Mentorship

Beyond formal mentorship, most participants also emphasized the critical role of informal mentorship in easing their transition into academic faculty groups. One wrote:

Informal mentors that I identified and befriended throughout the assimilation process ended up helping me more than anything else.

Several participants expressed the utility of informal mentorship in navigating departmental dynamics. One wrote:

Formal training didn’t address how to navigate departmental politics, but informal conversations with peers and mentors filled in the gaps.

Other areas participants found informal mentorship helpful included strategies for career advancement and promotion, academic prioritization, troubleshooting problems or unique situations on shift, “decoding cultural norms,” and learning about unwritten expectations or norms not formally communicated to faculty. Senior colleagues were the most frequently cited source of informal mentorship, although some also listed past colleagues, charge nurses, and other ancillary staff as sources of support and clarity. Participants suggested departments offer more opportunities for “informal ‘off the record’ candid discussions with senior or mid-career faculty,” citing “watercooler conversations” and social interactions as the most helpful informal channels that helped them learn insights not formally communicated.

#### Peer Guidance

While formal and informal mentorship provide crucial professional guidance and support, peer support offers a uniquely reciprocal and community-driven dynamic, fostering a sense of belonging, alleviating isolation, and addressing the emotional challenges of transitioning to an academic faculty role. “Connection with fellow junior faculty members” was frequently cited as the most helpful type of support during faculty transitions. One wrote, “Being lucky to find friends helping me navigate was my most important strength.” Some were surprised at their reliance on peers: “The most striking aspect of my transition was how much I had to rely on peers for guidance.” Participants consistently described the transition as overwhelming and isolating but emphasized that connecting with other junior faculty fostered camaraderie, facilitated advice exchange, and provided mutual support. Participants also found peer guidance useful in navigating topics such as salary transparency, project development, and clinical process clarity.

### Implicit Expectations/Norms

Numerous participants revealed a range of implicit expectations and norms encountered while assimilating into new academic environments (Table 2).

These expectations spanned various categories, including the nuanced role of mentorship, the importance of networking to establish professional relationships, navigating the balance between autonomy and collaboration within academic teams, and engaging in extracurricular activities to demonstrate commitment beyond clinical or teaching responsibilities. When asked to reflect on crucial unspoken insights and how they acquired this knowledge, participants wrote “many insights often come from informal channels” such as peers, mentors, informal social events, and through experiential and observational learning. While many supported making implicit norms more explicit through resources like guidebooks or presentations, they acknowledged that full acclimation ultimately comes through time and experience.

### Work-Life Integration

Participants identified work-life integration as a major challenge during the transition to faculty life (Table 3).

They described difficulty balancing clinical, teaching, research, administrative, and personal responsibilities, often citing a lack of preparation and unclear expectations. Subthemes included administrative burden, unspoken role expectations, competing demands, unequal clinical load, emotional labor, and the need for strategic time management. While some rspondents voiced concerns about long-term sustainability, others found fulfillment in the diversity of the role. Strategies such as prioritization, mentorship, and institutional support were noted as helpful in managing these pressures.

### Cultural Adjustment

Another key theme was cultural adjustment, or adapting to a department or institution’s unique culture and values, distinct from implicit expectations as it aimed to encompass broader institutional dynamics beyond individual roles. Many respondents found this to be the most challenging aspect of their transition, emphasizing the importance of navigating informal power dynamics and aligning with the cultural values of their institution. Others noted the unexpected influence of cultural norms on clinical practice, such as adjusting patient loads based on anticipated sign-out dynamics. One participant observed:

It took longer to integrate myself into the overall culture of the department, which is nuanced and constantly shifting.

Mentorship and emotional intelligence also played critical roles in facilitating cultural adjustment. Strategies for success included establishing good relationships with staff, admitting when one doesn’t know something, and applying lessons learned from residency. Some found familiarity in cultures that mirrored their training, while others underscored the dynamic nature of academic environments that require adaptability and patience to navigate. However, many recognized that no amount of preparation could fully capture the unique and ever-evolving nuances of each institution’s culture.

### Scholarly Output

Junior faculty consistently expressed stress about research and grant writing, frequently citing unclear expectations for scholarly output:

I encountered ambiguity around departmental expectations for research productivity.

And:

Unlike residency, there’s a greater expectation to contribute to the department’s academic output, which can feel daunting sometimes.

Navigating unclear expectations about publication frequency and the level of initiative required often depended on informal consultations with peers and senior faculty to clarify departmental norms. The emphasis on research output, sometimes perceptively valued over teaching, also added to the pressure. One faculty shared, “I am expected to produce double the publications per year that I was expecting.”

The stress of unclear research expectations was further compounded for many by an absence of formal training in essential skills such as study design, statistics, and grant writing. One wrote, “The research without formal training has been challenging.” Another suggested, “More formal support on research skills would help.” Despite these challenges, respondents acknowledged the potential of research as a vessel for career advancement, collaboration, and networking. One faculty noted the following:

Publishing papers often required collaboration with other researchers, and participating in joint projects or interdisciplinary teams was seen as a valuable way to build one’s academic reputation.

Many stressed the importance of protected time and institutional funding for research, for these greatly ease the challenge of balancing research with other professional responsibilities.

### Onboarding and Role Transparency

Onboarding and role transparency were identified as critical areas for improvement. Many respondents cited gaps in their orientation, unclear role expectations, and a lack of open communication during the onboarding process as significant challenges. One participant described, “Orientation lacked any practical guidance on managing academic priorities, navigating politics, or finding mentors.” This lack of clarity extended to areas such as patient flow, staff responsibilities, committee expectations, promotion pathways, reporting measures, and bonus structures, often leaving faculty to rely on informal channels and experiential learning for information. “There are definite expectations for ‘new’ attendings, especially junior ones, that are not mentioned. True ‘onboarding’ often occurs on the job through direct experience, because these things are difficult to communicate in an official manner.” Many described this approach as insufficient and advocated for clearer communication, greater transparency, and more structured onboarding. One participant urged,

Overhaul the onboarding process. Being more open and transparent from the get-go about all procedures. Nothing should be hidden.

Other suggestions included guidance on reporting structures, salary models, and advancement pathways, along with practical supports like shadow shifts and documented job responsibilities.

## DISCUSSION

This study highlights the role of the unwritten curriculum in shaping how junior faculty adapt to and navigate their new academic environments in EM. Unlike the hidden curriculum, which reflects broad institutional messaging, the unwritten curriculum identified in this study was characterized by situational, role-specific expectations that junior faculty described as needing to actively decode during early faculty transitions. While the results are presented thematically based on inductive coding, the findings align conceptually with Schlossberg’s transition theory. Themes related to clinical/academic workload and institutional expectations reflect the “situation” domain. Feelings of unpreparedness and imposter syndrome align with the “self” domain, while mentorship, onboarding, and peer support represent aspects of “support.” Strategies such as seeking peer mentorship or navigating unwritten expectations correspond to the “strategies” domain. This alignment reinforces the utility of Schlossberg’s framework in understanding junior faculty transitions, even when themes emerge inductively.

**A key finding of this study was the common experience of isolation and the common desire for a sense of belonging among new faculty**. Although we did not collect demographic data on race or ethnicity, prior studies have shown that minoritized faculty often face racism and disparities in development, promotion, and mentorship, contributing to greater feelings of isolation than their non-minoritized peers.^18^ Isolation has also been linked to imposter syndrome, which can impact work performance, clinical decision-making, and ultimately patient care and outcomes.^18^,^19^ Similarly, developing a sense of belonging has been recognized as a crucial factor in faculty engagement and career satisfaction across academic disciplines, highlighting its role in fostering collaboration, professional fulfillment, and overall retention.^20^–^22^ However, the barriers to faculty developing this sense of belonging have not been well explored.^20^

Given the centrality of belonging in early faculty experiences, institutions should take intentional steps to foster inclusive environments that reduce isolation. Structured onboarding programs should extend beyond logistics and compliance to include relational integration such as community-building sessions, faculty affinity groups, or cross-departmental peer networks. Additionally, **new faculty should be explicitly welcomed into the academic culture through introductions at meetings, early involvement in committees, and transparent conversations about institutional norms and expectations**. While such efforts may seem small, they are key in promoting psychological safety and cultivating a sense of professional identity and community.

Although most responses emphasized challenges and unmet needs, several participants also shared positive reflections on institutional or interpersonal support that helped facilitate their transition. These included the proactivity of department chairs in checking in with them, and their access to informal mentors, structured orientation programs, and strong peer communities. However, such examples were often framed as exceptions rather than standard practice, frequently described using terms such as “lucky” or “not everyone had this.” This pattern suggests that while effective structures do exist, they may be inconsistently implemented or inequitably distributed across departments.

Our findings suggest that **new faculty benefit most from structured mentorship that includes clear expectations, defined career milestones, and frequent feedback**. Given that responsibilities may shift rapidly within a faculty member’s first year, mentorship should be ongoing rather than episodic. Institutions should consider quarterly mentorship check-ins, structured six-month and one-year progress reviews, and faculty development programming tailored to early-career needs. Ideally, departments would offer incentives for mentorship participation; however, most academic hospital systems prioritize scholarship over citizenship within promotion criteria, making mentoring a lower priority.^23^ This imbalance can discourage faculty engagement in mentorship and exacerbate challenges in meeting academic expectations, particularly in scholarly productivity.

Because we did not systematically collect information on what formal structures were in place at each participant’s institution, it is difficult to determine whether certain supports (such as assigned mentors, regular check-ins, or orientation programs) were absent or simply experienced as ineffective. Several participants described having access to such structures “on paper” but lacking meaningful engagement or follow-through. Others noted that formal supports existed but felt this support was impersonal or misaligned with their actual needs. **Accordingly, our recommendations should be interpreted as highlighting variability in implementation rather than universal absence of support**.

Although mentorship was briefly referenced in one survey question and not formally defined, many participants used the term unprompted in their responses, indicating its salience in early faculty experiences. Their reflections suggest that mentorship needs to extend beyond traditional academic guidance to include role modeling, advocacy, and help interpreting institutional culture. National survey data highlight the same variability, with fewer than half of EM departments reporting formal mentoring programs, inconsistent structures and training, or recognizing the association of strong mentoring practices with greater scholarly productivity and National Institutes of Health funding.^24^ In our survey, participants described a wide range of mentorship experiences, reinforcing the need for **relationship-centered, longitudinal mentorship that supports both professional and personal development**.

Notably, some participants expressed a desire for senior mentors who shared similar clinical burdens, highlighting a tension between expectations and the reality of academic progression. **Rather than reflecting individual shortcomings, these perspectives underscore the importance of transparent mentorship conversations about role evolution, competing demands, and the multidimensional nature of academic success**.

While formal mentorship has long been recognized as a cornerstone of faculty development, our findings suggest that peer-support structures are equally critical in fostering professional belonging and resilience. Many participants described relying on peer support to bridge gaps left by formal orientation programs, with some considering themselves “lucky” to find supportive colleagues; this raises the question of whether faculty integration should be left to chance or whether institutions can take deliberate steps to foster meaningful connections. Our findings suggest that structured interventions, such as peer mentorship programs, faculty learning communities, and intentional networking opportunities, may help mitigate early-career isolation and improve professional satisfaction. Similar to our participants’ reliance on peer networks, prior qualitative work has shown that early professional socialization hinges less on formal orientation and more on the quality of senior colleagues’ interactions, with approachable and supportive behaviors from consultants fostering a safe learning environment while ineffective or dismissive behaviors undermine it.^25^ Peer mentorship can also be highly effective when new faculty mentor each other by sharing insights, fostering collaboration, strengthening relationships, and enhancing scholarly productivity.^26^ Peer-driven affinity groups and specialty-specific networks may further support early-career development.^27^

Another key finding was the challenge of meeting scholarly expectations, which raises questions about gaps in residency training, faculty development, or both. Many participants felt unprepared for research responsibilities in academia, suggesting deficiencies in residency research training. While barriers to scholarly productivity during residency are well studied, fewer studies have examined faculty challenges post-transition.^28^–^39^ Prior work suggests that structured research opportunities and institutional investment may improve preparedness for academic careers, although similar factors in EM remain unclear.^39^–^42^ **These findings support a dual approach that includes both improved research training during residency and structured faculty development addressing scholarly productivity and promotion expectations**.

Finally, many studies highlight the challenge of balancing work and home life and finding strategies to ease this burden.^43^^–48^ However, few respondents in our study mentioned difficulties integrating personal and professional responsibilities; instead, nearly all focused on the challenge of balancing academic and clinical duties. While we did not collect detailed family demographic data, this finding suggests that early-career stress for this cohort was driven primarily by competing professional demands, highlighting another dimension of the unwritten curriculum.

## LIMITATIONS

While our sample was geographically diverse, it may not fully represent the breadth of experiences across all academic EM programs, and institutional identifiers were not collected to preserve anonymity. Thus, we cannot confirm that all responses came from distinct institutions, although participants were recruited broadly and not intentionally sampled from the same sites. Most participants were personally known to at least one member of the research team, which may have introduced selection bias by oversampling individuals with similar professional interests or greater engagement with medical education scholarship.

Although all participants were within their first five years of academic practice, five held the rank of associate professor; their advanced rank may reflect prior experience or faster promotion and could have biased responses toward more successful transitions and greater familiarity with the academic environment. Neither did we elicit fellowship training status and faculty size were, which may influence comfort with academic expectations and access to mentorship.

The survey was administered in a fixed order, opening potential for respondent fatigue; however, analysis of character counts across open-ended responses showed no decline in response length over time. The final, mandatory, open-ended question under the “strategies” domain had a higher average character count (275.8) than both the first open-ended question in the survey (206.4) and the first domain-based question under “situation” (234.5), suggesting minimal evidence of fatigue-related decay. Some participants may have provided more concise answers than they would have in a verbal format, potentially limiting the depth of certain narratives.

Future research could build on our findings by exploring how faculty experiences evolve beyond their initial transition period and assessing whether structured mentorship and faculty development initiatives lead to measurable improvements in career satisfaction, scholarly productivity, and long-term retention.

## CONCLUSION

The findings of this study underscore the potential impact of the unwritten curriculum on junior faculty experiences in academic emergency medicine. Providing clear guidance, fostering structured mentorship, and supporting scholarly growth are essential to ensure that new faculty members thrive in their roles. Additionally, the disconnect between scholarly expectations and faculty preparedness highlights an area for improvement in both residency training and faculty development programs. Faculty success should not depend on luck or chance; by prioritizing mentorship, transparency, and structured career development, institutions can create an environment where junior academic faculty do not merely survive but rather excel early in their careers. Taken together, these findings function as a needs assessment that identifies actionable targets for improving onboarding and early faculty development in academic emergency medicine.

## Supplementary Information



## Figures and Tables

**Table 1:** Demographics of junior academic emergency medicine faculty who completed a survey as part of a study of the “unwritten curriculum”—a set of implicit norms, values, and expectations.

Demographic characteristics (N = 35)		
Gender identity		
Male	16	45.7%
Female	18	51.4%
Genderqueer	1	2.9%
Current age		
< 25	1	2.9%
25–30	13	37.1%
31–35	9	5.7%
36–40	11	31.4%
41–45	1	2.9%
Years in practice as attending physician		
1	6	17.1%
2	16	45.7%
3	4	11.4%
4	4	11.4%
5	5	14.3%
Institutional rank		
Clinical instructor	17	48.6%
Assistant professor	13	37.1%
Associate professor*	5	14.3%
Practice region		
New England (CT, ME, MA, NH, RI, VT)	5	14.3%
Mid-Atlantic (NJ, NY, PA)	13	37.1%
Midwest (IL, IN, IA, KS, MI, MN, MO, NE, ND, OH, SD, WI)	6	17.1%
South (AL, AR, DC, DE, FL, GA, KY, LA, MD, MS, NC, OK, SC, TN, TX, VA)	5	14.3%
West (AK, AZ, CA, CO, HI, ID, MT, NV, NM, OR, UT, WA, WY)	6	17.1%

* At some institutions, promotion to associate professor may occur after four years at the assistant professor rank, which explains the presence of associate professors within five years of residency completion.

**Table 2:** Junior emergency medicine faculty perceptions of implicit expectations and institutional norms during early academic transition.

What specific expectations or norms have you encountered while assimilating into a new academic culture that were not formally communicated to you?		
Sub-theme	Definition(s)	Supportive quotes
Mentorship	The importance of informal mentorship	“Mentorship also emerged as an essential part of the culture, though it was not always explicitly outlined. Senior faculty members often took a hands-on approach to providing guidance, but it was up to me to seek them out and foster those relationships.”
	Being available to mentor residents	“Constant availability for mentorship without clear acknowledgment or support”
Networking	Importance of networking to career advancement	“I found that collaborating with certain key individuals in the department significantly boosts your reputation, but this wasn't openly discussed.”
	Prioritization of informal events, not just formal ones	“I've learned that showing up to informal events and getting involved in the department's social life is just as important as the formal responsibilities. No one told me that being 'present' outside of the clinic or classroom would be so crucial for building connections and a solid reputation.” “It became clear that maintaining visibility by attending social events and informal gatherings was important for career progression.”
	Importance of networking in research	“I quickly learned that networking within the department is essential for securing research opportunities, something no one formally mentioned.” “Publishing papers often required collaboration with other researchers, and participating in joint projects or interdisciplinary teams was seen as a valuable way to build one’s academic reputation”
Balance between autonomy and collaboration	When to escalate/ask for help from a senior colleague and when to figure it out on your own	“Knowing when to approach senior faculty for advice and when to take the initiative” “Unspoken norms included balancing autonomy with collaboration” “There was an underlying emphasis on autonomy, especially in managing clinical responsibilities and research projects, while simultaneously being expected to seek help when needed (a balance that wasn’t clearly communicated up front)”
Extracurricular involvement	Taking on academic tasks and responsibilities not outlined in contract	“I noticed that I was expected to take on some academic tasks and responsibilities on my own initiative, even without explicit rules.”
	Taking on committee roles	“There was an unspoken expectation to take on additional committee roles to demonstrate involvement, which wasn't explicitly outlined during onboarding.”
	Demonstration of leadership	“There was an unspoken expectation to... demonstrate leadership, even if I was still adjusting to my role.”
	Availability to work on resident projects	“Being available to work with residents on projects”
	Increased involvement as junior faculty	“There's an unspoken understanding that junior faculty often take on more tasks to prove themselves, even though it wasn't spelled out.”
	Research	“The research component/expectations and what that looks like” “The new academic culture also attaches great importance to the practicality and application of academic research.”
Clinical care	Expected patient flow and expectations of attending vs other staff	“Expected patient flow. Expected responsibilities of the attending vs the other staff in terms of patient care - picking up supplies, cleaning the room, etc”
	Sign-out culture	“The sign-out culture is a big one.” “The hand-off culture and structure was not specified.”
	How to manage APNs	“The role of supervising APNs. I felt confused about how to manage with little guidance and nonexperience doing this previously. ”
	Number of patients per hour being higher than communicated	“At my new institution the volume of patients was higher than communicated.” “Expected number of patients to see per hour/day.”
	Attitude toward consultants, expectations for timely recommendations, and conflict mediation	“There is a much more laid-back attitude towards delays in consultant recommendations and how they communicate those recommendations.” “Delineated responsibilities between departments and who mediates disputes when informal discourse fails” “Culture regarding communication with surgical services (ie, attendings do not want to talk to ED attendings)”
	Senior resident/attending interactions and level of supervision	“Attending/resident relationship expectations” “Expectation from senior residents that I should not be ‘questioning’ their plans or focusing on teaching because they are prioritizing their speed and seeing the bulk of the patients in the department” “Lack of ownership of senior residents to take accountability to help teach and help their juniors on shift”
Department culture	Adapting to department’s culture	“Adapting to the department's unique culture, values, and expectations” “I discovered unspoken expectations to prioritize departmental loyalty over personal boundaries.” “Publication and academic presentations are most valued. This was odd to me, coming from a more clinically focused residency.”
	Norms about departmental politics	“Hierarchy and role of senior EM leadership within the ED” “There were unwritten rules about how to navigate the hierarchy.” “Who mediates disputes when informal discourse fails” “Informal channels for communicating with administrators”
Communication	Email etiquette, response times, and communication channels	“There's an implicit norm to respond promptly to emails, especially from senior faculty, even outside of standard working hours.”
Administrative	Pay structure and buy down	“I often have to cross-check my paycheck to make sure that things haven't been missed.” “The process of buy down”
	Pay differences	“There is often a pay gap in my shop between women and men.”
	Promotions	“How academic promotions work”
	Work habits and productivity	“Norms around work hours, flexibility, and productivity metrics”
	Scheduling and shift trade culture	“The schedule of academic activities is often flexible.” “Schedule requests” “At my current institution if you don’t request (off for a holiday) you likely won’t get a “vacation” around then because you’ll be doing the in-between days.” “People are constantly paying each other to swap shifts.”

**Table 3:** Work-life integration challenges experienced by junior academic emergency medicine faculty

Sub-theme	Definition	Supportive quotes
Role complexity	Juggling multiple expectations beyond clinical care	“Balancing clinical duties, teaching, and research feels overwhelming compared to the singular focus on patient care during residency.” “As a faculty member, the expectations shift significantly to include not only clinical practice but also a significant amount of teaching, research, service, and administrative work. The challenge lies in managing this balance. The pressure to excel in all these areas can be overwhelming, and there’s often no one-size-fits-all approach to finding that equilibrium.” “I quickly realized that the expectations for research, teaching, and administrative duties required a steep learning curve.” “Formal onboarding barely addressed the realities of juggling clinical work, academic expectations, and unspoken departmental norms…”
Administrative burden	Challenges with paperwork, evaluations, and institutional processes	“The most challenging aspect of being a faculty member compared to a resident is the increased administrative burden. As a faculty member, I have additional responsibilities like committee work, grant writing, and academic leadership roles. These responsibilities can be time-consuming and take away from clinical work and research.” “Managing administrative tasks and understanding institutional policies was never part of my training, making it a steep learning curve.” “It was challenging transitioning to this role, not just switching from a resident to faculty role but everything that comes with that—such as managing resident notes, filling out evaluations, participating in committees, putting out academic output for promotion, etc.”
Unspoken expectations	Unclear or evolving job scope	“There were unspoken expectations about how to balance clinical work, research, and teaching responsibilities.” “I think at first I was just going to be someone who came into work and went home—but the reality is, especially within academics, there is so much more expected of you that you don’t know until you start.”
Competing demands	Feeling pulled in many directions simultaneously	“It feels like there's always something else pulling my attention; and managing it all without neglecting any one part has been tough.” “Your attention is constantly being divided, yet you are still expected to be professional, teach residents and students, move the department to meet metric expectations, and escalate issues in a timely fashion. It’s an incredible amount of responsibility and pressure.” “The stress of being pulled in so many directions of patient care, medical student expectations, and often it feels like when you’re trying to do the right thing for patients it gets weaponized that you were unprofessional or crossed a line by asking questions.”
Strategic time use	Triaging and prioritizing non-clinical work amid clinical duties	“As time has progressed, I have a better understanding of what is essential to be completed in the short term, the near term, and the far term. This has helped me better triage what needs to get done now from a non-clinical perspective, which tends to be more porous RE deadlines. That skill is particularly important when I'm working a lot clinically and have little bandwidth.”
Clinical load disparities	Perceived higher workload for junior faculty	“Junior faculty were working more clinically and often being asked to take on more than their senior colleagues.”
Sustainability and fulfillment	Tension between burnout and professional satisfaction	“I just have recognized how hard it will be to sustain my career.” “While the workload can be demanding, I’ve grown to appreciate the diverse range of opportunities and challenges” …
Emotional labor	Navigating interpersonal stress and emotional resilience	“It wasn't just about the technical aspects of the role but understanding how to deal with the emotional and social pressures of academia—whether it's the challenge of balancing work and personal life, or learning how to respond to the stress and frustrations of colleagues and students.”
Support needs	Calls for institutional recognition and support	“A focus on work-life balance and acknowledgment of faculty contributions would also help [better support junior faculty].”
