# Supplementary material for: Unveiling Unwritten Curricula: Enhancing Junior Faculty Development in Academic Emergency Medicine
Source: West J Emerg Med. 2026 Jun 28;27(4):1048–61. doi: 10.5811/westjem.50856 (PMC13436674; doi:10.5811/westjem.50856)
Supplement: Supplementary file 1 [file wjem-27-1048-s001.docx]

**Appendix A.** Additional thematic findings reported by junior faculty in academic emergency medicine.

| Theme | Sub-theme | Supporting quotes |
| --- | --- | --- |
| Importance of networking | Building relationships through social events | “I’ve learned that showing up to informal events and getting involved in the department’s social life is just as important as the formal responsibilities. No one told me that being ‘present’ outside of the ED would be so crucial for building connections and a solid reputation.” |
|  | Collaboration and career advancement | “I quickly learned that networking within the department is essential for securing research opportunities, something no one formally mentioned.” |
|  | Social connection as an asset | “I think my ability to get along with people has helped me the most [in the transition].” |
| Learning through experience | Trial and error as a learning process | “I just learned through experience. I ran into roadblocks on shifts and asked someone else who was working or I made mistakes and had to correct them.” |
|  | Challenges of transitioning to independence | “Residency taught me how to swim, the transition to attending at a different institution was like getting thrown into a cold wave pool.” |
|  | Adapting to unspoken norms | “Some things are just unspoken norms that you eventually learn when you make a mistake or admit someone to the wrong service, but no one explicitly tells you until you figure it out yourself.” |
|  | Challenges of unstructured guidance | “The lack of structured guidance and support as faculty was frustrating. Unlike residency, there’s no safety net—just a vague expectation to ‘figure it out,’ often at the cost of personal well-being.” |
| Learning through observation | Gaining insight through observation | “I tried to take a step back and observe. But transitioning to a new department is hard.” |
|  | Navigating ambiguous situations | “I wasn’t sure how to navigate departmental politics during a heated meeting, so I observed how others responded.” |
|  | Practical learning from senior colleagues | “I learned how to manage my teaching responsibilities effectively by observing senior faculty in action.” |
| Feedback | Value of constructive feedback | “Having clear and constructive feedback from my department leadership helped me refine my goals and meet expectations.” |
|  | Value of timely feedback | “Regular teaching evaluation allows me to understand my teaching performance and adjust teaching strategies in time.” |
|  | Proactive feedback-seeking | “My ability to seek out and accept constructive feedback helped me refine my academic and clinical practices.” |
|  | Challenges with unclear feedback processes | “I wish there was a mandatory way to receive feedback here, but there wasn’t, and I had no idea what I was doing well and where I could improve.” |
| Professional confidence | Challenges in decision-making | “The most challenging thing about being an attending is that you make the final decision.” |
|  | Challenges with imposter syndrome | “I was surprised about how little I actually knew and how easy it was to question myself about the smallest things.” |
|  | Impact of unfamiliar dynamics on performance | “I second guessed myself unnecessarily, just because I was not familiar with the expected dynamics in this situation.” |
|  | Strategies for confidence-building | “Focusing on the best care for the patient and making it not about yourself.” |
|  | Visibility and vocal contributions | “I discovered later that being visible and vocal in meetings can significantly impact how others perceive your contributions.” |
| Formal professional development | Value of structured programs | “Access to professional development workshops focused on teaching and research skills was a major resource for my transition.” |
|  | Need for comprehensive resources | “The lack of a defined curriculum for faculty development means navigating unwritten norms and building my career path largely through my own efforts.” |
|  | Suggestions for improvement | “Providing resources on professional development, such as workshops on academic career paths, grant writing, leadership skills, and time management, could assist junior faculty in gaining confidence and finding their footing in areas not explicitly covered by formal training.” |
| Conflict resolution | Learning assertiveness | “Learning to be less timid about speaking up when things don’t seem right.” |
|  | Managing conflicts with consultants | “I had to call a consultant late at night who was rude -- I focused the call on my concern for giving the best care to the patient and made it not about myself.” |
|  | Growth through personal challenges | “I was far less tolerant of microaggressions after [a challenging year] and spoke up more in the moment rather than agonizing over small things after shift.” |
| Diversity, equity, and inclusion | Challenges of bias and microaggressions | “I found it especially challenging to gain respect as a woman of color. People approached other attendings before they approached me with things.” |
|  | Systemic inequities | “There is often a pay gap in my shop between women and men. I often feel underpaid for things and have to cross-check my paycheck to make sure that things haven’t been missed.” |
|  | Unwritten norms and power dynamics | “How do the staff treat female physicians or those of a diverse background? How do physicians treat each other?” |

**Appendix B.** Unveiling unwritten curricula survey.

**Appendix C.** Standards for Reporting Qualitative Research (SPQR) checklist.

O’Brien B.C., Harris, I.B., Beckman, T.J., Reed, D.A., & Cook, D.A. (2014). Standards for reporting qualitative research: a synthesis of recommendations. *Academic Medicine, 89(9)*, 1245-1251.

| No. Topic | Item | Page Number(s) |
| --- | --- | --- |
| Title and abstract |  |  |
| S1 Title | Concise description of the nature and topic of the study identifying the study as qualitative or indicating the approach (e.g., ethnography, grounded theory) or data collection methods (e.g., interview, focus group) is recommended | 1 |
| S2 Abstract | Summary of key elements of the study using the abstract format of the intended publication; typically includes objective, methods, results, and conclusions | 1 |
| Introduction |  |  |
| S3 Problem formulation | Description and significance of the problem/phenomenon studied; review of relevant theory and empirical work; problem statement | 2-3 |
| S4 Purpose or research question | Purpose of the study and specific objectives or questions | 2-3 |
| Methods |  |  |
| S5 Qualitative approach and research paradigm | Qualitative approach (e.g., ethnography, grounded theory, case study, phenomenology, narrative research) and guiding theory if appropriate; identifying the research paradigm (e.g., positivist, constructivist/interpretivist) is also recommended | 4 |
| S6 Researcher characteristics and reflexivity | Researchers’ characteristics that may influence the research, including personal attributes, qualifications/experience, relationship with participants, assumptions, or presuppositions; potential or actual interaction between researchers’ characteristics and the research questions, approach, methods, results, or transferability | 5-6 |
| S7 Context | Setting/site and salient contextual factors; rationale^a^ | 6-7 |
| S8 Sampling strategy | How and why research participants, documents, or events were selected; criteria for deciding when no further sampling was necessary (e.g., sampling saturation); rationale^a^ | 6-7 |
| S9 Ethical issues pertaining to human subjects | Documentation of approval by an appropriate ethics review board and participant consent, or explanation for lack thereof; other confidentiality and data security issues | 5 |
| S10 Data collection methods | Types of data collected; details of data collection procedures including (as appropriate) start and stop dates of data collection and analysis, iterative process, triangulation of sources/methods, and modification of procedures in response to evolving study findings; rationale^a^ | 3-5 |
| S11 Data collection instruments and technologies | Description of instruments (e.g., interview guides, questionnaires) and devices (e.g., audio recorders) used for data collection; if/how the instrument(s) changed over the course of the study | 3-5 |
| S12 Units of study | Number and relevant characteristics of participants, documents, or events included in the study; level of participation (could be reported in results) | 1, 6-7 |
| S13 Data processing | Methods for processing data prior to and during analysis, including transcription, data entry, data management and security, verification of data integrity, data coding, and anonymization/deidentification of excerpts | 7-8 |
| S14 Data analysis | Process by which inferences, themes, etc., were identified and developed, including researchers involved in data analysis; usually references a specific paradigm or approach; rationale^a^ | 7-8 |
| S15 Techniques to enhance trustworthiness | Techniques to enhance trustworthiness and credibility of data analysis (e.g., member checking, audit trail, triangulation); rationale^a^ | 7-8 |
| Results/findings |  |  |
| S16 Synthesis and interpretation | Main findings (e.g., interpretations, inferences, and themes); might include development of a theory or model, or integration with prior research or theory | 8-13 |
| S17 Links to empirical data | Evidence (e.g., quotes, field notes, text excerpts, photographs) to substantiate analytic findings | 8-13 |
| Discussion |  |  |
| S18 Integration with prior work, implications, transferability, and contribution(s) to the field | Short summary of main findings; explanation of how findings and conclusions connect to, support, elaborate on, or challenge conclusions of earlier scholarship; discussion of scope of application/generalizability; identification of unique contribution(s) to scholarship in a discipline or field | 15-20 |
| S19 Limitations | Trustworthiness and limitations of findings | 13-15 |
| Other |  |  |
| S20 Conflicts of interest | Potential sources of influence or perceived influence on study conduct and conclusions; how these were managed | 6 |
| S21 Funding | Sources of funding and other support; role of funders in data collection, interpretation, and reporting | 6 |

^a^The rationale should briefly discuss the justification for choosing that theory, approach, method, or technique rather than other options available, the assumptions and limitations implicit in those choices, and how those choices influence study conclusions and transferability. As appropriate, the rationale for several items might be discussed together.
